# Detection of Latent Tuberculosis Infection in Patients with Rheumatological Diseases Who Receive Immunosuppressive Therapy

**DOI:** 10.3390/diagnostics16121883

**Published:** 2026-06-17

**Authors:** Anna Starshinova, Adilia Sabirova, Alexey Maslyanskiy, Irina Grigorieva, Raul Sharipov, Ravil Tukfatullin, Aleksandr Panteleev, Michail Nazarenko, Dmitry Kudlay

**Affiliations:** 1 Almazov National Research Medical Center, 197341 St. Petersburg, Russia; 2 Department of Mathematics and Computer Science, Saint Petersburg State University, 199034 St. Petersburg, Russia; 3 Medical Department, Bashkir State Medical University of the Ministry of Health of the Russian Federation, 450008 Ufa, Russia; 4 Institute of Medicine, Department of Pathology, St. Petersburg State University, 199034 St. Petersburg, Russia; 5 Medical Department, St. Petersburg State Medical University Named after Academician I.P. Pavlov, 197022 St. Petersburg, Russia; 6 St. Petersburg Research Institute of Phthisiopulmonology, 190961 St. Petersburg, Russia; 7 St. Petersburg State Budgetary Healthcare Institution “City Tuberculosis Dispensary”, 196142 St. Petersburg, Russia; 8 Department of Pharmacology, Institute of Pharmacy, I.M. Sechenov First Moscow State Medical University, 119991 Moscow, Russia; 9 FSBI SSC Institute of Immunology FMBA of Russia, 115522 Moscow, Russia; 10 Faculty of Bioengineering and Bioinformatics, Lomonosov Moscow State University, 119991 Moscow, Russia

**Keywords:** latent tuberculosis infection, risk groups, immunodiagnostics, recombinant tuberculosis antigen test, IGRA, rheumatological diseases, immunosuppressive therapy

## Abstract

**Background/Objectives:** Latent tuberculosis infection (*LTBI*) is a persistent immune response to *Mycobacterium tuberculosis* antigens in the absence of clinically active tuberculosis. It is now established that progression from *LTBI* to active tuberculosis is directly associated with immune dysregulation, which frequently occurs in immune-mediated diseases requiring treatment with immunosuppressive agents. The aim of this study was to identify *LTBI* in patients with rheumatological diseases receiving immunosuppressive therapy using contemporary immunodiagnostic methods. **Materials and Methods:** A retrospective, prospective, group-control study was conducted, analyzing the results of immunodiagnostics in patients with rheumatological diseases on immunosuppressive therapy and without established contact with tuberculosis patients (*n* = 44; main group). The control group consisted of healthy individuals (*n* = 51) with no history of tuberculosis contact, clinical or radiological manifestations of the disease, or signs of chronic pathology exacerbation. Both groups were predominantly female (72.7% in the main group and 62.8% in the control group). The mean age in the patient group was 49.1 years (95% *CI* [44.77; 53.43]). Rheumatoid arthritis was diagnosed in 29.6% (13) of patients (95% *CI* [16.06; 43.03]). Articular syndrome was observed in at least 72.7% (32) of patients (95% *CI* [59.57; 85.89]). In 54.6% (24) of cases (95% *CI* [39.83; 69.26]), patients received biologic immunosuppressive therapy as basic treatment. In 15.9% (7) of cases (95% *CI* [5.10; 26.72]), patients received conventional synthetic disease-modifying antirheumatic drugs (*DMARDs*). Of these, 71.43% (5) (95% *CI* [35.24; 92.44]) underwent comprehensive examination to exclude active tuberculosis prior to biologic therapy initiation. For immunodiagnostics, all subjects underwent an interferon-gamma release assay (*IGRA*) and/or testing with a recombinant tuberculosis antigen (*ATR*) sample, with dynamic assessment of test results. All patients with positive immunodiagnostic results underwent multidetector computed tomography of the chest organs. The level of *LTBI* in the comparison groups was defined as the percentage of positive immunological test results at a significance level of *p* < 0.05. Statistical data processing was performed using Microsoft Excel 2019. **Results:** In the main group, positive immunodiagnostic results were recorded in 20.5% (9) of cases (95% *CI* [8.54; 32.37]), which is significantly higher than in the control group of healthy individuals (5.8% (3), 95% *CI* [1.41; 16.54]). This reflects statistically significant differences in immunological test results between groups receiving and not receiving immunosuppressive therapy (χ^2^ = 4.545, *p* = 0.034). Dynamic evaluation of the *ATR* sample revealed positive results in 21.7% (5) of cases (95% *CI* [4.88; 38.60]), with four out of five patients demonstrating positive conversion. In the third assessment, positivity was observed in 33.33% (5) of cases (95% *CI* [9.48; 57.19]), which was higher than in the first (χ^2^ = 1.025, *p* = 0.312) and second assessments (χ^2^ = 0.629, *p* = 0.428), although these differences were not statistically significant. Notably, in two out of five patients, the *ATR* test result changed from negative to positive. **Conclusions:** In patients with rheumatological diseases receiving immunosuppressive therapy, *LTBI* was detected in 20.5%, which is significantly higher than in healthy individuals (5.8%, *p* = 0.034). Furthermore, there was an increase in the proportion of positive tests over time (up to 21.7% and 33.3% on immunotherapy), suggesting an increasing risk of progression to active tuberculosis infection.

## 1. Introduction

Latent tuberculosis infection (LTBI) is defined as a state of persistent immune response caused by the presence of *Mycobacterium tuberculosis* (MTB) antigens in the host organism in the absence of clinical manifestations of active tuberculosis [1]. According to a number of studies, the lifetime risk of developing active tuberculosis in individuals infected with MTB is approximately 5–10%, most commonly occurring within the first five years following primary infection [2].

It is now well established that progression from LTBI to active tuberculosis is closely associated with immune regulation, which is frequently impaired in immune-mediated diseases as well as during immunosuppressive therapy administered for comorbid conditions [3,4]. Individuals with altered immune status are therefore classified as high-risk groups for tuberculosis, as outlined by the World Health Organization in its guidelines published in 2014 and updated in 2018 [1].

In recent years, genetically engineered biological agents, also referred to as biological disease-modifying antirheumatic drugs (bDMARDs), have played a major role in the treatment of immune-mediated diseases characterized by dysregulated or excessive immune responses, including rheumatic diseases. These therapies are particularly important in severe or refractory cases where remission cannot be achieved with conventional synthetic DMARDs (csDMARDs) alone [5,6]. However, the use of bDMARDs is associated with an increased risk of infectious complications, including tuberculosis, partly due to the development of immunosuppression and conditions such as neutropenia, which predispose to bacterial infections [7,8,9].

It should also be noted that the risk of bacterial infections, including pneumonia, is elevated in patients receiving csDMARDs; however, this risk is further increased with the use of biological agents [10].

Epidemiological data from the Russian Federation demonstrate a consistently high incidence of diseases of the musculoskeletal system and connective tissue, including rheumatic diseases, with rates of 30.0 in 2015, 29.4 in 2016, 29.3 in 2017, 29.6 in 2018, 30.1 in 2019, 24.8 in 2020, 26.4 in 2021, 28.7 in 2022, 31.2 in 2023, and 32.5 per 100,000 population in 2024 [11]. These trends suggest a likely increase in the use of bDMARDs alongside conventional immunosuppressive therapies, thereby necessitating strict adherence to screening and monitoring strategies in patients receiving such treatments in order to prevent the development of active tuberculosis [12].

Furthermore, due to long-term immunosuppressive therapy, patients with neurological autoimmune diseases, such as multiple sclerosis and myasthenia gravis, are also at increased risk of LTBI reactivation and require careful monitoring by tuberculosis specialists [13,14].

According to international studies, the prevalence of LTBI among patients receiving biological therapy is relatively high. For example, in Slovakia, the prevalence of LTBI prior to initiation of anti-cytokine therapy was low (7.25%), comparable to that of the general population; however, during treatment, it increased to 21.7% [8]. In Turkey, an endemic tuberculosis setting, positive conversion on the QuantiFERON-TB Gold In-Tube test during treatment with IL-17 inhibitors (secukinumab and ixekizumab) was observed in 10 patients with psoriasis [15]. Among patients with systemic vasculitis, LTBI was detected in 1.4% using ELISPOT [16], while in China, the prevalence of LTBI among patients with systemic lupus erythematosus reached 15% [17].

In the Russian Federation, Mordyk et al. (2024) reported an LTBI prevalence of 19.23% based on recombinant tuberculosis allergen testing in patients with rheumatic diseases receiving bDMARDs [8]. Additionally, Frolova and Borisov (2020) demonstrated a 6.3% rate of positive conversion of LTBI tests following initiation of anti-TNF therapy in patients with inflammatory bowel disease [17]. Positive immunological test results are therefore considered predictors requiring closer clinical monitoring.

Thus, immunological screening in patients with rheumatic diseases—both prior to initiation of immunosuppressive therapy and during treatment—plays a crucial role in tuberculosis control. This approach enables early detection of LTBI, informs clinical decision-making, and helps identify contraindications to certain immunosuppressive agents.

The aim of this study was to detect latent tuberculosis infection in patients with rheumatic diseases receiving immunosuppressive therapy using modern immunodiagnostic methods.

## 2. Materials and Methods

**Figure 1:**
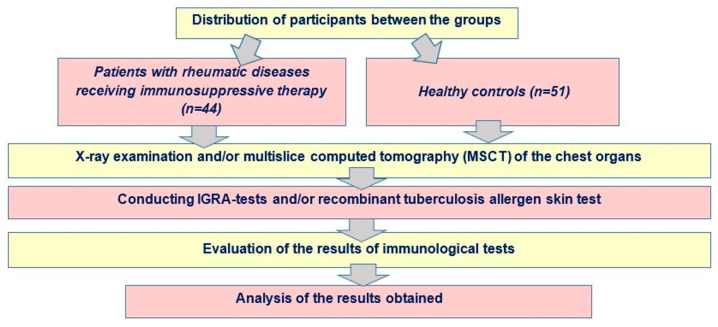
Study design.

A retrospective–prospective case–control study (2024–2026) was conducted to analyze the results of immunodiagnostic testing in patients with rheumatic diseases receiving immunosuppressive therapy (*n* = 44, study group) (Figure 1).

The retrospective component included analysis of clinical and laboratory data obtained prior to initiation of immunosuppressive therapy, whereas the prospective component included longitudinal follow-up with repeated immunodiagnostic assessment during treatment.

### 2.1. Inclusion Criteria

1. Male and female patients aged 18 to 75 years (inclusive);
2. Patients with rheumatic diseases receiving immunosuppressive therapy.

### 2.2. Exclusion Criteria

1. Age < 18 or >75 years;
2. Presence of primary immunodeficiency;
3. HIV infection;
4. Exacerbation of chronic diseases, including diabetes mellitus, malignancies, and other severe comorbid conditions;
5. Patients undergoing hemodialysis;
6. Pregnant or breastfeeding women;
7. Individuals with suspected active tuberculosis, confirmed tuberculosis, or a history of tuberculosis;
8. Known contact with a patient with tuberculosis.

The control group consisted of healthy individuals (*n* = 51) with no history of contact with tuberculosis patients and no clinical, radiological, or laboratory evidence of acute or chronic diseases in the active phase. Healthy controls were recruited from the same geographic region and represented a generally comparable urban population; however, exact matching for socioeconomic characteristics was not performed.

The distribution of participants between the groups was performed in accordance with the study design.

Women predominated in both groups (Table 1), accounting for 72.7% (95% CI: 59.57–85.89) in the study group and 62.8% (95% CI: 49.48–76.01) in the control group. The mean age was 49.1 years (95% CI: 44.77–53.43) in the study group and 29.2 years (95% CI: 26.79–31.61) in the control group. Given the substantial age difference between the groups, additional age-adjusted analyses were performed to reduce potential confounding effects.

### 2.3. Structure of Rheumatic Diseases

The structure of rheumatic diseases is presented in Table 2. Rheumatoid arthritis of varying activity predominated, accounting for 29.6% (*n* = 13; 95% CI: 16.06–43.03). Plaque psoriasis was observed in 18.2% (*n* = 8; 95% CI: 6.79–29.58). Juvenile rheumatoid arthritis and psoriatic arthritis each accounted for 9.1% (*n* = 4; 95% CI: 0.60–17.59). Less frequent conditions included ankylosing spondylitis, microscopic polyangiitis, psoriatic spondylitis, Sjögren’s syndrome, and unspecified arthritis, collectively accounting for 11.7% (*n* = 5; 95% CI: 1.99–20.74).

### 2.4. Clinical Characteristics and Therapy

At least 72.7% of patients (*n* = 32; 95% CI: 59.57–85.89) presented with articular syndrome, including arthralgia, morning stiffness, joint swelling, reduced mobility, and joint deformities. Skin manifestations (plaques, rashes, xerosis) were observed in 36.4% (*n* = 16; 95% CI: 22.15–50.58), while 2.3% (*n* = 1; 95% CI: 0.06–12.02) exhibited photosensitivity, mucosal dryness, and Raynaud’s phenomenon.

Biological therapy (bDMARDs) was administered in 54.6% of cases (*n* = 24; 95% CI: 39.83–69.26). Among these, secukinumab was used in 16.8% (*n* = 4), olokizumab in 12.5% (*n* = 3), and rituximab in 12.5% (*n* = 3), while other biological agents were used in the remaining cases.

Conventional synthetic DMARDs were used in 15.9% of patients (*n* = 7; 95% CI: 5.10–26.72), including methotrexate, mycophenolate mofetil, hydroxychloroquine, and leflunomide, as well as intra-articular injections and topical glucocorticoids. Among these patients, 71.43% (*n* = 5) underwent extended tuberculosis screening prior to initiation of biological therapy.

In 29.6% of cases (*n* = 13), data on baseline therapy were unavailable due to incomplete medical records.

Patients also received symptomatic and supportive therapy, including non-steroidal anti-inflammatory drugs (NSAIDs), intra-articular analgesics, topical dermatological agents, calcium supplements, and general supportive treatment. All patients were regularly followed up by a rheumatologist, and in some cases by a dermatologist. Follow-up intervals were generally standardized and included routine reassessment every 6–12 months; however, some variability occurred depending on clinical status, treatment regimen, and organizational aspects of routine clinical care.

One patient (2.3%) with juvenile rheumatoid arthritis developed infiltrative pulmonary tuberculosis with cavitation and bacterial excretion during long-term treatment with tocilizumab. Biological therapy was discontinued, and anti-tuberculosis chemotherapy for drug-susceptible tuberculosis was initiated. This patient was included retrospectively, with analysis of clinical data prior to tuberculosis onset. Baseline immunological screening results in this patient were negative before initiation of biologic therapy; subsequent conversion of immunological testing was observed during follow-up prior to the development of cavitary tuberculosis.

### 2.5. Diagnostic Procedures

All participants underwent:

- Detailed clinical assessment and medical history collection;
- Chest radiography;
- Immunological testing.

Immunodiagnostic evaluation included:

- Interferon-gamma release assay (IGRA; QuantiFERON-TB Gold);
- Recombinant tuberculosis allergen skin test (Diaskintest^®^, Russia).

In the study group, 14 patients underwent only IGRA (QuantiFERON-TB Gold) testing, while 30 underwent only the skin test. In the control group, 18 individuals underwent both tests, and 33 underwent only the skin test. The use of different immunodiagnostic methods reflected real-world clinical practice and depended on test availability, physician preference, contraindications to skin testing, and organizational considerations. In some cases, only one diagnostic method was available at the time of patient evaluation.

A strict procedural requirement was that blood sampling for the IGRA (QuantiFERON-TB Gold) test was performed prior to administration of the skin test.

In a subset of patients, immunodiagnostic results were assessed longitudinally. Conversion of IGRA results was defined as a transition from a negative to a positive result according to manufacturer-defined threshold criteria exceeding borderline variability values. Conversion of skin test results was defined as either the appearance of a positive reaction or a clinically significant increase in induration size according to national tuberculosis screening guidelines.

Interpretation of test results was performed in accordance with the manufacturer’s instructions. Laboratory personnel performing IGRA analyses followed standardized procedures, while skin test interpretation was conducted according to national clinical recommendations. The study was performed in a real-world clinical setting; therefore, full blinding to clinical data was not feasible.

Interpretation of test results was performed in accordance with the manufacturer’s instructions. Patients with positive results underwent additional evaluation, including multislice computed tomography (MSCT) of the chest, to exclude active tuberculosis. No radiological findings suggestive of tuberculosis were identified.

### 2.6. Statistical Analysis

Statistical analysis and graphical visualization were performed using Microsoft Excel 2019, Statistica 8.0 (StatSoft, Tulsa, OK, USA), and GraphPad Prism 4.00 (GraphPad Software Inc., San Diego, CA, USA).

The prevalence of LTBI was defined as the proportion of positive immunological test results (%) with a significance level of *p* < 0.05. Comparisons between groups were performed using Pearson’s χ^2^ test. Given the substantial age difference between the study groups, exploratory age-adjusted analyses were performed. These analyses suggested that the observed association between immunosuppressive therapy and LTBI prevalence remained statistically significant. However, due to the limited sample size and heterogeneity of clinical subgroups, formal multivariable modeling was not considered statistically robust and was therefore not performed.

Confidence intervals (95% CI) for continuous variables (age) were calculated using the one-sample Student’s *t*-test following descriptive statistics estimation. For binomial variables, confidence intervals were calculated using Wald’s normal approximation.

A 95% confidence level was applied throughout. Confidence intervals were graphically represented using box plot functions in Microsoft Excel.

## 3. Results

In the group of patients with rheumatic diseases receiving immunosuppressive therapy, positive IGRA and/or recombinant tuberculosis allergen skin test results were identified in 20.5% of cases (*n* = 9; 95% CI: 8.54–32.37). Among positive cases, positive IGRA results were observed in 20.5% (*n* = 9), whereas positive recombinant allergen skin test results were observed in 5.8% (*n* = 3). This proportion was significantly higher than that observed in healthy controls (5.8%, *n* = 3; 95% CI: 1.41–16.54), indicating a statistically significant difference between groups (χ^2^ = 4.545, *p* = 0.034) (Table 3).

**Figure 2:**
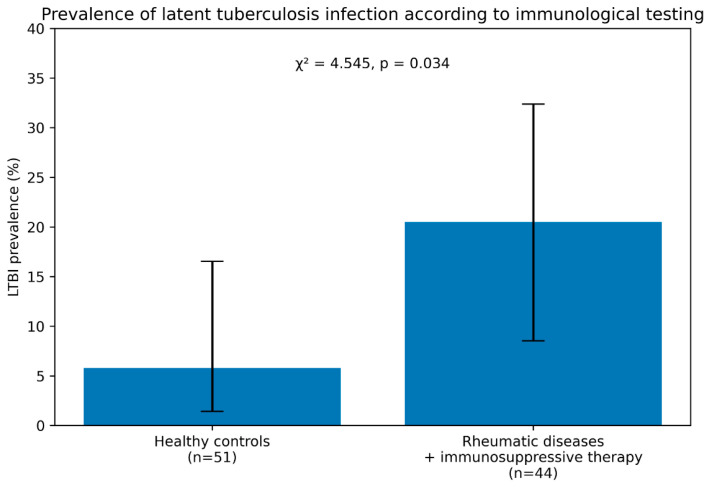
Prevalence of latent tuberculosis infection (LTBI) according to immunological testing in the study groups. LTBI prevalence based on positive IGRA and/or recombinant tuberculosis allergen skin test results was significantly higher among patients with rheumatic diseases receiving immunosuppressive therapy (20.5%; 95% CI: 8.54–32.37) than among healthy controls (5.8%; 95% CI: 1.41–16.54) (χ^2^ = 4.545, *p* = 0.034). Error bars indicate 95% confidence intervals.

The proportion of positive results (20.5%) reflects the prevalence of latent tuberculosis infection (LTBI) in patients with rheumatic diseases and significantly exceeds that observed in healthy individuals (Figure 2).

Given that immunodiagnostic results were assessed longitudinally in a subset of patients with rheumatic diseases, the following findings were obtained (Table 4).

At the second assessment, positive results of the recombinant allergen skin test were observed in 21.7% of patients (*n* = 5; 95% CI: 4.88–38.60), which was slightly higher than at baseline; however, the difference was not statistically significant (χ^2^ = 0.015, *p* = 0.903). Among these patients, 80% (*n* = 4; 95% CI: 35.96–97.97) demonstrated conversion from a negative to a positive result, while in 20% (*n* = 1; 95% CI: 2.03–64.04) the result remained positive at both time points.

At the third assessment, positive results were recorded in 33.3% of patients (*n* = 5; 95% CI: 9.48–57.19), which was higher than both the initial (χ^2^ = 1.025, *p* = 0.312) and second assessments (χ^2^ = 0.629, *p* = 0.428); however, these differences were also not statistically significant. Among these patients, 40% (*n* = 2; 95% CI: 11.60–77.09) showed conversion from negative to positive compared with the second assessment, 40% (*n* = 2) had already been positive at the second assessment, and 20% (*n* = 1; 95% CI: 2.03–64.04) remained positive across all three assessments.

**Figure 3:**
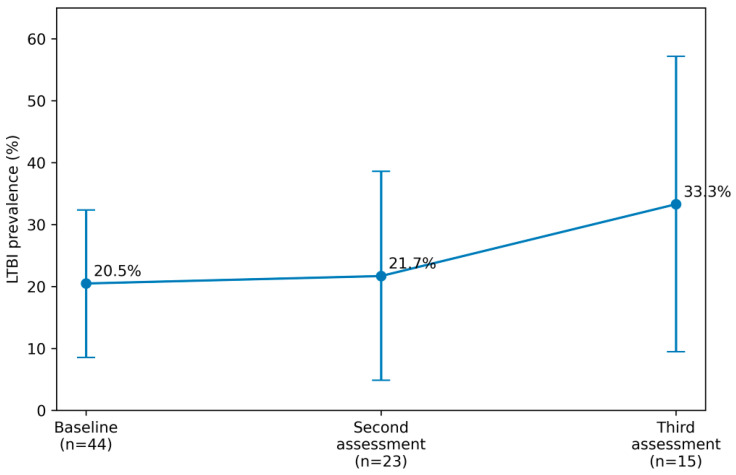
Longitudinal assessment of latent tuberculosis infection (LTBI) prevalence in patients with rheumatic diseases receiving immunosuppressive therapy. LTBI prevalence increased from 20.5% (95% CI: 8.54–32.37) at baseline to 21.7% (95% CI: 4.88–38.60) at the second assessment and 33.3% (95% CI: 9.48–57.19) at the third assessment. Error bars represent 95% confidence intervals. Differences between assessments were not statistically significant.

Thus, a gradual increase in LTBI prevalence was observed among patients with rheumatic diseases receiving immunosuppressive therapy over time, rising from 20.5% at baseline to 21.7% and subsequently to 33.3% (Figure 3).

Overall, the prevalence of LTBI based on immunological testing in patients with rheumatic diseases receiving immunosuppressive therapy was significantly higher (20.5%) compared with healthy individuals (5.8%). Longitudinal assessment demonstrated a progressive increase in LTBI prevalence to 21.7% and subsequently to 33.3%, although these changes did not reach statistical significance.

## 4. Discussion

The prevalence of latent tuberculosis infection (LTBI) among patients with rheumatological diseases, predominantly represented by rheumatoid arthritis (29.6%), was 20.5%, which clearly indicates an increased risk of progression to active tuberculosis compared with healthy individuals from the general population (5.8%).

Rheumatoid arthritis is one of the most common chronic autoimmune diseases in Europe, with a prevalence of approximately 0.8–1.1%. Patients with rheumatoid arthritis are at increased risk of developing active tuberculosis in the presence of LTBI due to both long-term immunosuppressive therapy and underlying immune dysregulation. The use of targeted therapies may contribute to adverse hematological effects, including neutropenia, thereby increasing susceptibility to bacterial infections. Several cohort and retrospective studies have demonstrated a higher prevalence of LTBI among patients with rheumatoid arthritis compared with the general population, with some reports indicating up to a fourfold increase in tuberculosis incidence.

A study conducted in Slovakia assessed LTBI prevalence, including the use of interferon-gamma release assays (IGRAs), in patients with moderate-to-severe rheumatoid arthritis. The prevalence of LTBI prior to initiation of anti-cytokine therapy was low (7.25%) and comparable to that of the general population; however, during treatment, LTBI was detected in 21.7% of patients. These findings highlight the necessity for enhanced tuberculosis surveillance in patients with rheumatoid arthritis, particularly those receiving biologic therapies. In the present study, the LTBI prevalence of 20.5% observed in patients receiving immunosuppressive therapy—including both conventional disease-modifying antirheumatic drugs and biologic agents—is consistent with the 21.7% reported by Malinová et al. (2021) [9].

In recent years, the effectiveness of rheumatological disease management has significantly improved with the introduction of targeted therapies. However, due to their effects on key cytokines and immune regulation, the risk of infections—including viral, bacterial, fungal, and mycobacterial—remains a major concern. A study conducted in Saudi Arabia involving 410 patients receiving adalimumab, etanercept, or tocilizumab found no significant association between these agents and an increased risk of tuberculosis. Only 0.3% of patients receiving adalimumab and 0.9% receiving etanercept demonstrated IGRA conversion during therapy [18]. Nevertheless, it cannot be excluded that baseline immunosuppression may have influenced the observed lack of conversion.

Compared with tumor necrosis factor-alpha (TNF-α) inhibitors, anti-IL-17A agents are generally considered to carry a lower risk of LTBI reactivation or progression to active tuberculosis. In a tuberculosis-endemic setting in Turkey, patients with psoriasis receiving secukinumab or ixekizumab for more than 12 months were evaluated using the QuantiFERON-TB Gold In-Tube assay [14]. LTBI conversion was observed in 10 out of 334 initially IGRA-negative patients. While positive IGRA results do not confirm active tuberculosis, they may indicate the need for closer clinical monitoring.

Similarly, Hu et al. (2025) [19] evaluated 306 patients with psoriasis receiving IL-17A inhibitors in China. Among 220 patients who were initially IGRA-negative, 17 converted to IGRA-positive status during follow-up, and one case of active tuberculosis was reported. Increased interferon-gamma levels were observed in both initially IGRA-negative and IGRA-positive patients, suggesting that the risk of LTBI persists despite therapy [19].

Systemic vasculitis represents another autoimmune condition associated with an increased risk of tuberculosis. In a multicenter study involving 191 patients, LTBI was detected in 31.4% of cases using the ELISPOT assay [15]. For comparison, a large population-based cohort study in rural China reported an LTBI prevalence of approximately 20.3% among individuals aged ≥15 years [20].

These findings suggest that the interpretation of LTBI screening results in patients with rheumatological diseases should not be limited to binary test outcomes but should also account for the patient’s immunological status. Thus, test sensitivity depends not only on assay performance but also on host immune reactivity.

Another important challenge is the discordance between different immunological tests. A systematic review by Pyo et al. (2018), including 5224 patients with rheumatic diseases, demonstrated positivity rates of 29% for the tuberculin skin test, 17% for QuantiFERON-TB Gold In-Tube, and 18% for ELISPOT Agreement rates between the tuberculin skin test and IGRA methods ranged from 73% to 75%, indicating that reliance on a single diagnostic method may be insufficient [21]. These findings support the rationale for combined immunodiagnostic strategies in immunocompromised patients. The dual-testing approach used in the present study is generally consistent with several international recommendations advocating enhanced LTBI surveillance in high-risk populations receiving biologic or targeted immunosuppressive therapy, although international guidelines differ regarding routine implementation of combined testing strategies.

Immunological assays may also be useful for LTBI detection in patients with inflammatory bowel disease despite substantial immunosuppression. In one study, a positive ATP-based test result was observed in 3.7% of patients, compared with 28.8% for the tuberculin skin test, with a concordance coefficient of 0.37. When only strongly positive reactions were considered, concordance increased to 0.62. These findings further support the need for combined diagnostic approaches due to potential discordance between tests [22].

In the Russian Federation, research and the clinical implementation of these diagnostic tests commenced in 2012. Russian scientists can be regarded as pioneers in the development of in vivo methods for diagnosing latent tuberculosis infection. In particular, a research team from the Research Institute of Molecular Medicine at the I.M. Sechenov Moscow Medical Academy, led by Academician M.A. Pal’tsev and Corresponding Member of the Russian Academy of Sciences V.I. Kiselev, was the first to develop a novel skin test based on a recombinant tuberculous allergen (Diaskintest). The successful clinical evaluation of this test was carried out as early as 2008. The test incorporates the antigens *ESAT-6* and *CFP-10*, which are absent in *Mycobacterium bovis BCG* strains. This ensures a high degree of specificity and enables differentiation between *BCG*-induced hypersensitivity and true infection with *Mycobacterium tuberculosis*. This represents a significant methodological advantage over the traditional tuberculin skin test, especially in *BCG*-vaccinated populations [23].

Clinical studies have demonstrated that the specificity of *Diaskintest* lies within the confidence interval of 90–100%, with no evidence of cross-reactivity due to prior *BCG* vaccination and no documented nonspecific allergic reactions. Furthermore, administration of the test at a dose of 0.2 µg in 0.1 mL elicits a delayed-type hypersensitivity reaction in 98–100% of individuals with active tuberculosis or confirmed *M. tuberculosis* infection [24,25].

Longitudinal monitoring of patients with rheumatological diseases using immunological assays provides clinically relevant information regarding immune responses to persistent tuberculosis during immunosuppressive therapy. In the present study, longitudinal follow-up demonstrated cases of immunological conversion prior to the development of active tuberculosis, supporting the importance of serial screening in high-risk patients receiving biologic therapy. A multidisciplinary approach involving both rheumatologists and tuberculosis specialists, along with structured patient management pathways, appears to be a justified strategy aimed at reducing the risk of tuberculosis development, progression, and associated comorbidity burden.

### 4.1. Study Limitations

Several limitations of this study should be acknowledged. First, the relatively small sample size, particularly in the longitudinal subgroup, may limit the statistical power to detect significant differences over time. This limitation also restricts the robustness of multivariable statistical analyses and limits the generalizability of the findings. The absence of microbiological confirmation of tuberculosis infection restricts the ability to distinguish between true infection, reactivation, and immunological test variability. Potential confounding factors, such as prior Bacillus Calmette–Guérin (BCG) vaccination, environmental exposure, and heterogeneity of immunosuppressive regimens, were not fully controlled [26].

Furthermore, the use of immunological assays alone may not fully reflect the complex host–pathogen interactions underlying LTBI [27,28]. Future studies incorporating molecular and immunological biomarkers could provide a more comprehensive understanding of tuberculosis risk in this population.

### 4.2. Clinical Implications and Future Directions

The results of this study have important clinical implications. Regular LTBI screening should be considered a mandatory component of the management of patients with rheumatological diseases prior to and during immunosuppressive therapy. Particular attention should be paid to patients receiving biologic agents, especially TNF-α inhibitors.

Future research should focus on:

- Large-scale prospective studies to validate LTBI screening strategies;
- Development of more sensitive and specific diagnostic tools;
- Identification of immunological and genetic biomarkers predicting LTBI reactivation;
- Optimization of preventive therapy protocols in immunocompromised patients.

## 5. Conclusions

The prevalence of LTBI, as determined by test IGRA and Diaskintest - based immunological testing, was 20.5% among patients with rheumatological diseases receiving immunosuppressive therapy, which is significantly higher than that observed in healthy individuals (5.8%). Longitudinal assessment demonstrated an increase in LTBI prevalence to 21.7% at the second evaluation and to 33.3% at the third evaluation; however, these differences did not reach statistical significance. These findings indicate that regular LTBI screening using immunological assays in patients with rheumatological diseases undergoing immunosuppressive therapy enables timely identification of LTBI and supports optimization of clinical management strategies.

## Figures and Tables

**Table 1:** Composition of the study groups by gender and age.

Group	Men, % (*n*)	95% CI	Women, % (*n*)	95% CI	Mean Age	95% CI
Patients with rheumatic diseases receiving immunosuppressive therapy (*n* = 44)	27.3 (12)	14.11–40.43	72.7 (32)	59.57–85.89	49.1	44.77–53.43
Healthy controls (*n* = 51)	37.3 (19)	23.99–50.52	62.8 (32)	49.48–76.01	29.2	26.79–31.61

**Table 2:** Structure of rheumatic diseases.

Disease	% (*n*)	95% CI
Rheumatoid arthritis	29.6 (13)	16.06–43.03
Plaque psoriasis	18.2 (8)	6.79–29.58
Juvenile rheumatoid arthritis	9.1 (4)	0.60–17.59
Psoriatic arthritis	9.1 (4)	0.60–17.59
Systemic lupus erythematosus	4.6 (2)	0.42–15.97
Scleroderma	4.6 (2)	0.42–15.97
Other	11.7 (5)	1.99–20.74
Data unavailable	6.8 (3)	1.68–18.89

**Table 3:** Results of immunodiagnostic testing in the study groups.

Group	Positive Results % (*n*)	95% CI	Negative Results % (*n*)	95% CI	χ^2^	*p*-Value
Patients with rheumatic diseases receiving immunosuppressive therapy (*n* = 44)	20.5 (9)	8.54–32.37	79.5 (35)	67.63–91.46	4.545	0.034
Healthy controls (*n* = 51)	5.8 (3)	1.41–16.54	94.2 (48)	83.46–98.59	–	–

Critical χ^2^ Value = 3.841, *p* < 0.05.

**Table 4:** Dynamics of immunodiagnostic results in patients with rheumatic diseases receiving immunosuppressive therapy.

Assessment	Positive Results % (*n*)	95% CI	Negative Results % (*n*)	95% CI
Initial assessment (*n* = 44)	20.5 (9)	8.54–32.37	79.5 (35)	67.63–91.46
Second assessment (*n* = 23)	21.7 (5)	4.88–38.60	78.3 (18)	61.40–95.12
Third assessment (*n* = 15)	33.3 (5)	9.48–57.19	66.7 (10)	42.81–90.52

## Data Availability

The datasets generated and/or analyzed during the current study are available from the corresponding author on reasonable request.
